# Stanniocalcin-1 Protects Retinal Ganglion Cells by Inhibiting Apoptosis and Oxidative Damage

**DOI:** 10.1371/journal.pone.0063749

**Published:** 2013-05-07

**Authors:** Sang Jin Kim, Jung Hwa Ko, Ji-Hyun Yun, Ju-A Kim, Tae Eun Kim, Hyun Ju Lee, Seok Hwan Kim, Ki Ho Park, Joo Youn Oh

**Affiliations:** 1 Department of Ophthalmology, Samsung Medical Center, Sungkyunkwan University School of Medicine, Gangnam-gu, Seoul, Korea; 2 Clinical Research Center, Samsung Biomedical Research Institute, Gangnam-gu, Seoul, Korea; 3 Department of Ophthalmology, Seoul National University Hospital, Jongno-gu, Seoul, Korea; 4 Laboratory of Ocular Regenerative Medicine and Immunology, Biomedical Research Institute, Seoul National University Hospital, Jongno-gu, Seoul, Korea; 5 Department of Ophthalmology, Seoul National University Boramae Hospital, Dongjak-gu, Seoul, Korea; University of Florida, United States of America

## Abstract

Optic neuropathy including glaucoma is one of the leading causes of irreversible vision loss, and there are currently no effective therapies. The hallmark of pathophysiology of optic neuropathy is oxidative stress and apoptotic death of retinal ganglion cells (RGCs), a population of neurons in the central nervous system with their soma in the inner retina and axons in the optic nerve. We here tested that an anti-apoptotic protein stanniocalcin-1 (STC-1) can prevent loss of RGCs in the rat retina with optic nerve transection (ONT) and in cultures of RGC-5 cells with CoCl_2_ injury. We found that intravitreal injection of STC-1 increased the number of RGCs in the retina at days 7 and 14 after ONT, and decreased apoptosis and oxidative damage. In cultures, treatment with STC-1 dose-dependently increased cell viability, and decreased apoptosis and levels of reactive oxygen species in RGC-5 cells that were exposed to CoCl_2_. The expression of HIF-1α that was up-regulated by injury was significantly suppressed in the retina and in RGC-5 cells by STC-1 treatment. The results suggested that intravitreal injection of STC-1 might be a useful therapy for optic nerve diseases in which RGCs undergo apoptosis through oxidative stress.

## Introduction

Optic neuropathy is a disease of axons of retinal ganglion cells (RGCs) in the optic nerve, and is one of the leading causes of irreversible visual loss [1], [2]. The causes for axonal damage in the optic nerve are diverse ranging from neurodegenerative and neuroinflammatory diseases to glaucoma that affects more than 60 million people around the world and causes bilateral blindness in about 8 million people [3]. The final pathway of diverse forms of optic neuropathies is the death of RGCs occurring mainly through apoptosis [2], and the generation of reactive oxygen species (ROS) takes an intrinsic part in RGC apoptosis [4]–[6]. Similar to other mammalian neurons in the central nervous system, axons and RGCs are unable to regenerate, and thus no therapeutic treatment is available to date for optic neuropathies.

Stanniocalcin-1 (STC-1) is a 247 amino acid protein that is secreted from cells as a glycosylated homodimer. STC-1 was originally identified as a calcium/phosphate regulatory protein in fish [7]. Although its physiological function in humans is not clear, STC-1 is physiologically active in mammals and may be involved in regulation of cellular calcium/phosphate homeostasis [8]. In addition, mammalian STC-1 has been shown to have multiple biological effects involving protection of cells against ischemia [9], [10], suppression of inflammatory responses [11], or reduction of ROS and the subsequent apoptosis in alveolar epithelial cancer cells [12] and photoreceptors in the retina [13]. Also, it was found that STC-1 was secreted by mesenchymal stem cells (MSCs) in response to signals from apoptotic cells and mediated an anti-apoptotic action of MSCs [14].

Here we investigated the effects of STC-1 on the apoptosis of RGCs and on ROS production in the retina of rats with intraorbital optic nerve transection (ONT), a well-established model for optic neuropathy that induces rapid and specific RGC degeneration and results in apoptotic death of more than 80% of RGCs within 2 weeks [15]. In addition, we evaluated the STC-1 effect in cultures of RGCs with CoCl_2_ injury that causes RGC apoptosis by several mechanisms including ROS-driven oxidative stress [16], [17].

## Materials and Methods

### Ethics Statement

The animal study was performed in strict accordance with the Association for Research in Vision and Ophthalmology Statement for the Use of Animals in Ophthalmic and Vision Research. The experimental protocol was approved by the Institutional Animal Care and Use Committee of Samsung Medical Center (SMR112051).

### Animals and animal model

Eight-week-old male Sprague-Dawley rats weighing 200 to 250 g were purchased from Orient Bio Inc. (Seongnam, Korea), and used in all experiments. Under anesthesia with zolazepam-tiletamine (Zoletil®, Virbac, Carros, France) and xylazine, the pupils were dilated with phenylephrine/tropicamide eyedrops, and transection of optic nerve was performed as previously described [18], [19]. Briefly, after exposing an optic nerve through a superotemporal conjunctival incision, optic nerve sheath was incised longitudinally, and cross-section of the optic nerve was made at 2 mm from the eyeball with a 20-gauge MVR blade. Immediately after ONT, preservation of blood supply to the optic nerve head and the retina was confirmed by fundus examination, and the rats received an intravitreal injection of either 2 µL STC-1 (1 μg) or the same volume of PBS using a Hamilton syringe with a 33 gauge needle (Hamilton, Reno, NV). Recombinant human STC-1 was purchased from BioVender (Brno, Czech Republic). According to the manufacturer's instructions, distilled water was added to a vial of STC-1 that was lyophilized in 20 mM Tris buffer, 20 mM NaCl to yield a final solution of 0.5 mg/mL, and sterilized through a filter before use. The rats were sacrificed at days 1, 7, and 14, and the retinas were subjected to analysis. Eyes with postoperative complications such as cataract or infection were excluded from analysis.

### Determination of RGC density

For retrograde labeling of surviving RGCs, the fluorescence tracer dextran tetramethylrhodamine (DTMR; 3,000 MW, Molecular Probes Inc., Eugene, OR) was applied to the proximal surface of transected optic nerve as previously described [18], [19]. DTMR diffuses passively through the axon toward the cell soma at a rate of 2 mm/h which subsequently label the surviving retinofugal RGCs with a competent axon [19], [20]. At days 1, 7, 14, and 28, eyeballs were enucleated and fixed in 4% paraformaldehyde for 4 h. The retinas were isolated from eyeballs, and four cuts were made from the edges to the center of the retina. The retinas were then flattened and mounted vitreous side up on slide glasses and covered with fluorescent mounting media (Dako, Glostrup, Denmark). The whole-mounted retinas were observed under a laser confocal microscope (LSM700; Carl Zeiss MicroImaging GmbH, Jena, Germany), and images were acquired at 100× magnification. The density of labeled RGCs was determined by counting cells in the fields 1, 2, and 3 mm from the center of the optic nerve along the centerline of each retinal quadrant. The number of labeled cells in a total of 12 photographs was divided by the area of the region and pooled to calculate the mean density of labeled cells per square millimeter for each retina. The numbers of RGCs were counted independently by two observers in a masked fashion, and averaged.

### Cell culture

For an *in vitro* study, we used RGC-5 cells, a transformed rat RGC line that has been well-characterized as cells expressing ganglion cell markers and exhibiting ganglion cell-like behavior [21]. The cells were a kind gift from Dr. N. Agarwal [19]. Cells were cultured in Dulbecco's minimal essential medium (DMEM) containing 4500 mg/L glucose, 10% heat-inactivated fetal bovine serum, and 1% penicillin/streptomycin in a humidified incubator with 5% O_2_ at 37°C. When 70% confluence was reached, the cells were exposed to CoCl_2_ (100–800 μM; Sigma-Aldrich Co. LLC, St. Louis, MO) to induce hypoxia and apoptosis and treated with recombinant STC-1 (1–500 ng/mL; BioVender) or *N*-Acetyl-L-cysteine (Sigma). We used N-acetylcysteine as one of controls because a previous report showed that N-acetylcysteine protected RGC-5 cells from hypoxia-induced cell death by scavenging ROS [22].

### Assays for cell viability and apoptosis

Cell viability and proliferation were measured using MTT assay following the manufacturer's protocol (Vybrant® MTT Cell Proliferation Assay Kit; Invitrogen, Carlsbad, CA). Apoptosis was measured by flow cytometry (FACSCanto flow cytometer; BD BioSciences, Mountain View, CA) after double-staining cells with propidium iodide (PI)-PE and annexin V-FITC (Molecular Probes, Inc., Leiden, The Netherlands). The populations of PI^+^ Annexin-V^+^ cells were compared between groups.

### ELISAs

For protein extraction, the retinas or the cells were sonicated on ice in tissue extraction reagent (Invitrogen) containing protease inhibitor cocktail (Roche, Indianapolis, IN). After centrifugation at 12,000 rpm at 4°C for 20 min, the supernatant was assayed for caspase-3 activity (Caspase-3/CPP32 colorimetric assay kit, Biovision, Milpitas, CA), nitrotyrosine content (OxiSelectTM Nitrotyrosine ELISA Kit, Cell Biolabs, Inc. San Diago, CA), protein carbonyl content (OxiSelectTM Protein Carbonyl ELISA Kit, Cell Biolabs, Inc.), or uncoupling protein 2 (UCP2; Rat Mitochondrial uncoupling protein 2 ELISA kit, Cusabio®, Wuhan, China).

### Western blot

Clear lysates of protein from the retinas or the cells were prepared as described above and measured for the concentration. A total of 50 µg protein was fractionated by SDS-PAGE on 10% bis-tris gel (Invitrogen), transferred to nitrocellulose membrane (Invitrogen), and then blotted with antibodies against HIF (hypoxia-inducible factor)-1α (Santa Cruz Biotechnology, Inc., Dallas, TX) or β–actin (Santa Cruz Biotechnology).

### Real time RT-PCR

For RNA extraction, the cells or the retinas were lysed in RNA isolation reagent (RNA Bee, Tel-Test Inc., Friendswood, TX) and total RNA was then extracted using RNeasy Mini kit (Qiagen, Valencia, CA). Double-stranded cDNA was synthesized by reverse transcription (SuperScript III, Invitrogen). Real-time amplification was performed (Taqman Universal PCR Master Mix, Applied Biosystems, Carlsbad, CA) and analyzed on an automated instrument (7500 Real-Time PCR System, Applied Biosystems). PCR probe sets were commercially purchased (Taqman Gene Expression Assay Kits, Applied Biosystems). Values were normalized to 18s RNA and expressed as fold changes relative to normal retinas or uninjured cells.

### Flow cytometrical analysis of mitochondrial ROS

Mitochondrial ROS was measured in cultures using CellROX^TM^ Deep Red Reagent (Invitrogen), a novel cell-permeant dye that fluoresces (near-infrared) when oxidized and MitoTracker Green FM Dye (Invitrogen), a probe that stains mitochondrial membrane lipid regardless of mitochondrial membrane potential. The cells were treated with CellROX™ dye and MitoTracker Green dye, and analyzed by flow cytometry (FACSCanto flow cytometer).

### Statistical analysis

The data are presented as the mean ± SEM. Comparisons of two values were made using the two-tailed Student's *t* test, and comparisons of more than two values using a one-way ANOVA (SPSS 12.0; SPSS software, Chicago, IL). Differences were considered significant at p<0.05.

## Results

### Intravitreal injection of STC-1 increased the survival of RGCs after ONT

**Figure 1:**
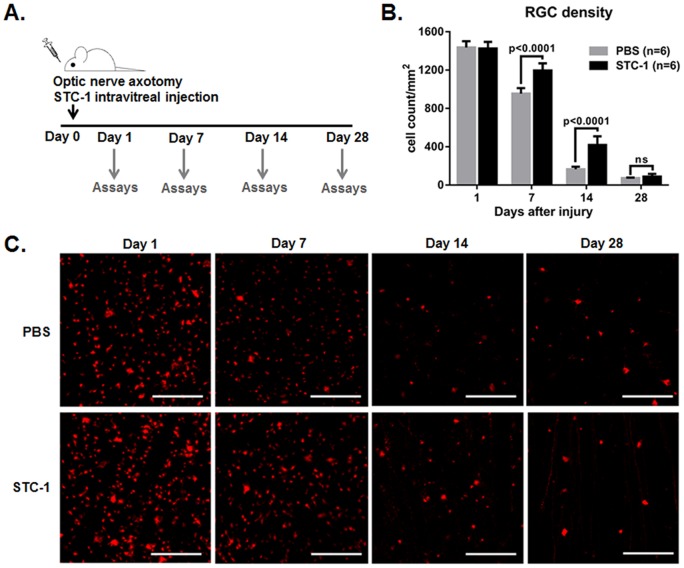
Intravitreal administration of STC-1 increased the survival of retinal ganglion cells after optic nerve transection. (**A**) Immediately after optic nerve transection, 1 μg STC-1 or PBS was injected into the vitreous cavity of rats, and the retinas were evaluated for retinal ganglion cells (RGCs) at days 1, 7, 14, and 28. (**B**) The density of RGCs was significantly higher in the retinas treated with STC-1 compared to PBS-treated retinas at all time-points examined as counted by cells retrogradely labeled with DTMR dye (**C**). The values are presented as the mean ± SEM. Scale bars, 100 μm.

To evaluate the effect of STC-1 on survival of RGCs *in vivo*, we injected 1 μg STC-1 into the vitreous cavity of rats immediately after ONT. At days 1, 7, 14, and 28, the rats were sacrificed, and the retinas were evaluated for RGCs (**Fig. 1A**). The numbers of RGCs at days 7 and 14 were significantly greater in rats that received STC-1 compared to controls that received PBS (**Fig. 1B, C**); the numbers of RGCs were 1196±30/mm^2^ in STC-1-treated rats and 955±23/mm^2^ in PBS-treated rats (p<0.0001) at day 7, and 419±36/mm^2^ in STC-1-treated rats and 166±10/mm^2^ in controls (p<0.0001) at day 14. There was no difference in the numbers of surviving RGCs between the groups at day 28 after ONT.

### STC-1 decreased apoptosis and oxidative damage in the retina after ONT

**Figure 2:**
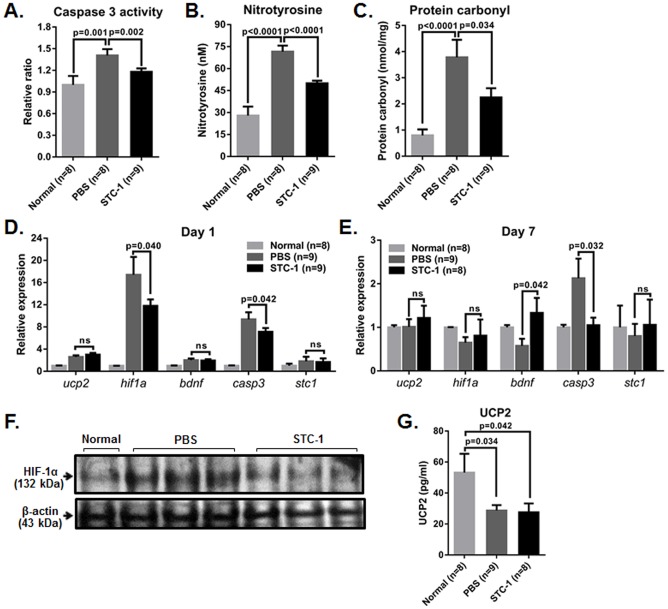
Intravitreal STC-1 administration decreased apoptosis and oxidative damage in the retina after optic nerve transection. (**A–C**) ELISA analysis showed that levels of active caspase-3 and two markers for oxidative damage (nitrotyrosine and protein carbonyl) were significantly decreased in the retina by an intravitreal injection of STC-1. (**D, E**) Real time RT-PCR indicated that levels of transcripts for HIF-1α and caspase-3 were increased in the retinas at day 1 after injury and significantly decreased by STC-1 treatment. The expression of caspase-3 was also significantly lower in the STC-1-treated retinas at day 7. (**F**) Western blot analysis confirmed that the expression of HIF-1α protein was increased in the retina at day 1 after ONT, and decreased by STC-1 injection. (**G**) The protein levels of UCP2 in the retina were decreased by ONT and not changed by STC-1 treatment. The values are presented as the mean ± SEM.

To investigate that STC-1 improved RGC survival by decreasing apoptosis, we analyzed the retina for the level of active caspase-3. Caspase-3 is implicated in the primary and secondary waves of RGC apoptosis and active for a long period of time and with a great intensity during RGC loss [23], [24]. As shown in **Fig. 2A**, caspase-3 activity at day 1 was significantly lower in the retinas of rats that received STC-1 compared to controls, indicating reduction of apoptosis by STC-1. Next, we assayed the retinas for nitrotyrosine and protein carbonyl, two protein derivatives of ROS that are used to measure oxidative damage in the retina [25], [26]. We evaluated ROS levels because previous studies reported that bursts of ROS were generated following ONT and triggered RGC apoptosis [2], [4]–[6]. The levels of both nitrotyrosine and protein carbonyl in the retinas at day 1 were significantly lower in STC-1-treated eyes compared to PBS-injected controls (**Fig. 2B, C** **)**.

### STC-1 decreased the expression of HIF-1α in the retina after ONT

Next, we used real time RT-PCR to evaluate the expression of oxidative stress- and apoptosis-related genes that are implicated in oxidative damage, RGC apoptosis, and survival: UCP2, HIF-1α, BDNF (brain-derived neurotrophic factor), and caspase-3 [2]. Additionally, we assayed for the expression of STC-1 to check whether ONT induced up-regulation of endogenous STC-1 in the retina because previous studies reported that STC1 transcript was increased in the heart or brain following hypoxic signals [27], [28]. The expression of all the genes tested increased at day 1 and decreased at day 7 after ONT (**Supplementary** **Fig. 1** **,** **Fig. 2D, E**). Of note, transcript levels of HIF-1α, a key regulator of hypoxia, were markedly increased in the retina at day 1, and were significantly reduced by intravitreal injection of STC-1 (**Fig. 2D**). Consistently, western blot analysis showed that levels of HIF-1α protein were increased in the retina at day 1 and markedly decreased in the retina treated with STC-1 (**Fig. 2F**). Also, levels of caspase-3 transcripts that were increased by ONT were significantly decreased by STC-1 at days 1 and 7 (**Fig. 2D, E**). However, the expression of UCP2 that was previously shown to be up-regulated by STC-1 [11], [29] was not increased in STC-1-treated retinas either at mRNA or protein levels (**Fig. 2D, E, G**). Also, STC1 transcripts were not increased in the retina after ONT and not altered by exogenous STC-1 treatment (**Supplementary** **Fig. 1** **,** **Fig. 2D, E**). The level of BDNF, that exerts a potent neuroprotective effect on RGCs *in vivo* and *in vitro* [30], [31], was significantly higher in the retinas of STC-1-treated eyes at day 7 compared to PBS-treated controls (**Fig. 2E**).

### STC-1 inhibited apoptosis in CoCl_2_-injured RGC-5 cells

**Figure 3:**
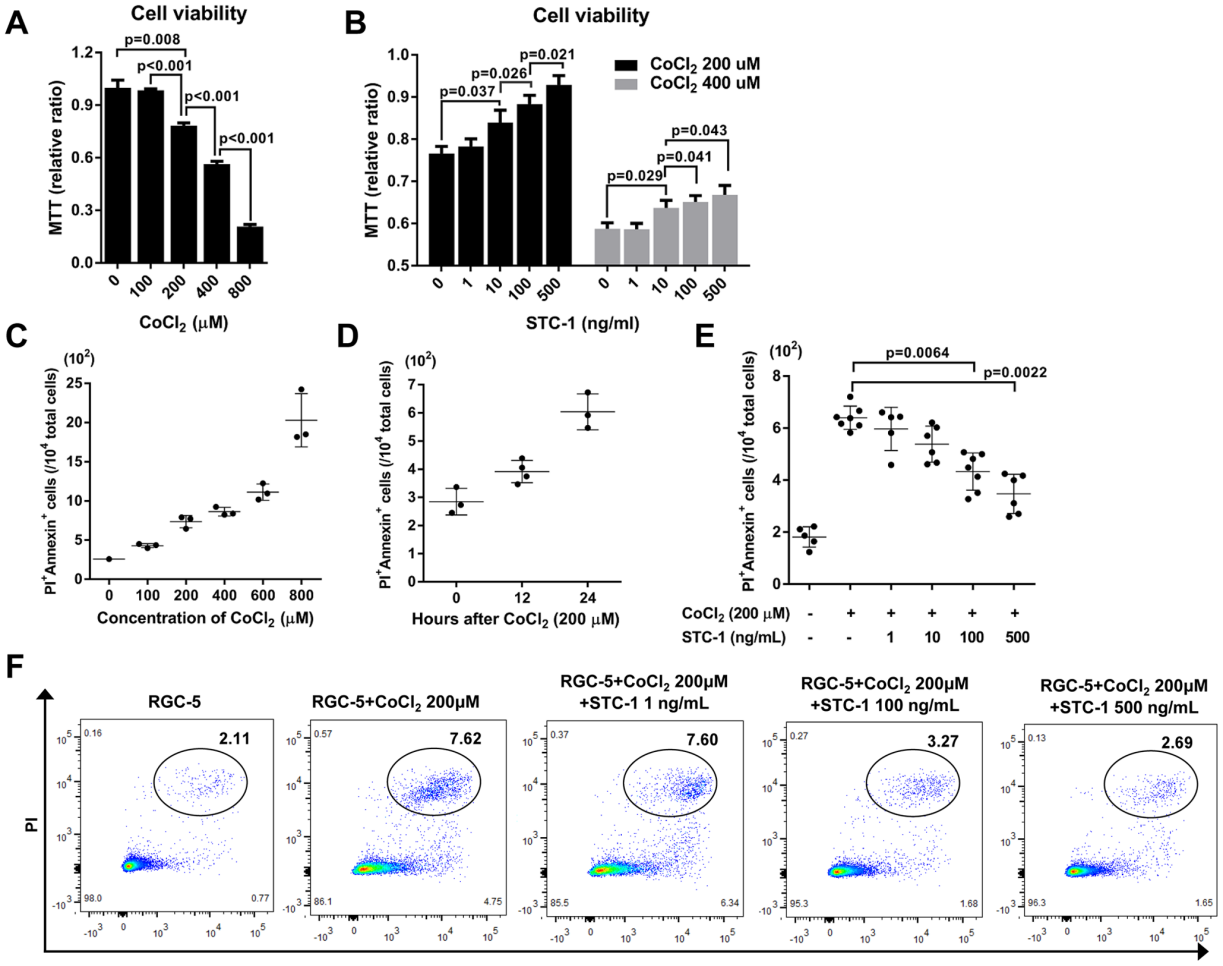
STC-1 inhibited apoptosis of RGC-5 cells exposed to CoCl_2_. (**A**) MTT assay showed that exposure to CoCl_2_ for 12 h decreased the viability of RGC-cells in a concentration-dependent manner. (**B**) STC-1 treatment significantly rescued RGC-5 cells that were injured by either 200 μM or 400 μM CoCl_2_ in a dose-dependent manner. (**C, D**) Flow cytometry showed that CoCl_2_ increased the numbers of PI^+^Annexin^+^ cells in RGC-5 cells in concentration- and time-dependent manners. (**E, F**) Both 100 and 500 ng/mL of STC-1 decreased the numbers of PI^+^Annexin^+^ cells in RGC-5 cells injured by 200 μM CoCl_2_. The values are presented as the mean ± SEM.

To evaluate the effect of STC-1 on the survival of RGCs *in vitro*, we exposed RGC-5 cells to different concentrations of CoCl_2_ (0–800 μM) for 12 or 24 h in order to induce hypoxia and apoptosis. Expectedly, CoCl_2_ decreased the cell viability, and STC-1 treatment significantly increased the cell viability in a dose-dependent manner as measured by MTT assay (**Fig. 3A, B**). Also, flow cytometry showed that the numbers of PI^+^Annexin^+^ cells indicating apoptotic cells were increased in RGC-5 cells after CoCl_2_ exposure in concentration and time-dependent manners (**Fig. 3C, D** **)**. Treatment with either 100 or 500 ng/mL STC-1 significantly decreased the numbers of PI^+^Annexin^+^ cells as assayed by flow cytometry (**Fig. 3E, F**).

### STC-1 suppressed CoCl_2_-induced ROS production and HIF-1α expression in RGC-5 cells

**Figure 4:**
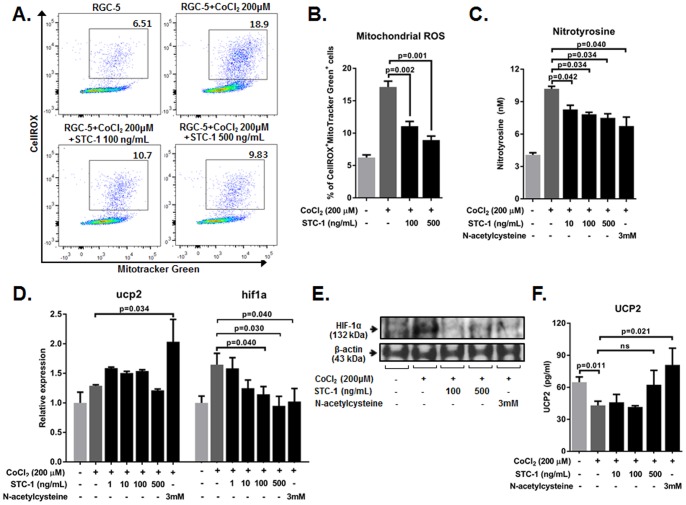
STC-1 inhibited ROS levels in RGC-5 cells exposed to CoCl_2_. (**A, B**) Flow cytometry showed that CoCl_2_ significantly increased the percentage of cells positive for both CellROX^TM^ and MitoTracker Green, a marker for oxidative stress, and treatment with either 100 or 500 ng/mL of STC-1 significantly decreased the percentages of CellROX^+^ MitoTracker Green^+^ cells in RGC-5 cells. (**C**) ELISA analysis for nitrotyrosine indicated that levels of nitrotyrosine were markedly increased in CoCl_2_-injured RGC-5 cells, and significantly decreased by STC-1 or N-acetylcysteine treatment. (**D**) Real time RT-PCR analysis indicated that expression of HIF-1α was induced in RGC-5 cells by CoCl_2_, and was significantly down-regulated by STC-1 (100 or 500 ng/mL). However, UCP2 transcripts were not increased by STC-1. (**E**) Western blot analysis for HIF-1α showed that HIF-1α protein was increased in RGC-5 cells after CoCl_2_ injury, and was decreased by STC-1 treatment. (**F**) ELISA showed that the levels of UCP2 protein were not increased in CoCl_2_-injured RGC-5 cells by STC-1 treatment, whereas N-acetylcysteine treatment significantly increased levels of UCP2. The values are presented as the mean ± SEM.

We next evaluated the effect of STC-1 on ROS production in RGC-5 cells that were exposed to CoCl_2_. The percentage of cells that were both positive for CellROX^TM^ and MitoTracker Green indicating production of mitochondrial ROS was increased by CoCl_2_, and reduced significantly by STC-1 treatment (**Fig. 4A, B**). Similarly, levels of nitrotyrosine, a marker of oxidative stress, were markedly increased in the cells by CoCl_2_ and significantly decreased by STC-1 or N-acetylcysteine (**Fig. 4C**). Together, data suggested that hypoxia induced by CoCl_2_ increased oxidative stress in RGC-5 cells, and STC-1 decreased oxidative stress. Also, similar to *in vivo* data (**Fig 2D, E, F**), the expression of HIF-1α was induced in RGC-5 cells by CoCl_2_ and significantly reduced by STC-1 both at transcript and protein levels (**Fig. 4D, E**). However, STC-1 treatment did not change the expression of UCP2 either at transcript or protein levels in RGC-5 cells, whereas N-acetylcysteine significantly increased UCP2 levels (**Fig. 4D, F**).

## Discussion

Data demonstrated that intravitreal injection of STC-1 delayed RGC apoptosis in a rat model of ONT. Also, treatment with STC-1 decreased CoCl_2_-induced apoptosis in RGC-5 cells. Both *in vivo* and *in vitro*, the anti-apoptotic effect of STC-1 was accompanied by decreases in ROS and by down-regulation in HIF-1α.

HIF-1 is a heterodimeric transcription factor that is composed of α and β subunits. HIF-1 acts as a key regulator for the cellular response to hypoxia [32]. Under normoxic condition, HIF-1α, the active subunit, is rapidly degraded by the ubiquitin-proteosome system. However, under hypoxic condition, HIF-1α is accumulated and facilitates apoptosis by activating diverse genes for pro-apoptotic proteins such as BNIP3 as well as stabilizing p53 which in turn activates genes to initiate apoptosis [33], [34]. In fact, high levels of HIF-1α were detected in the retina and optic nerve head of patients with glaucomatous optic neuropathy, indicating the involvement of hypoxia and HIF-1α in the pathogenesis of the disease [35], [36]. However, HIF-1α can also inhibit apoptosis by activating anti-apoptotic genes such as VEGF and Bcl-xL [37], [38]. Therefore, the role of HIF-1α on cell apoptosis is more complicated depending on the type of tissues and injuries. In our study, HIF-1α expression was down-regulated in STC-1-treated retinas and cell. These findings might be direct effects of STC-1 or indirect results of STC-1-mediated tissue protection reflecting that decreased damage in STC-1-treated tissues might reduce activation of HIF-1α in response to tissue damage. Therefore, HIF-1α might not be directly related to RGC damage or to the action of STC-1. Further studies are necessary to investigate the role of HIF-1α in RGC apoptosis and protection as well as potential implication of STC-1-induced down-regulation of HIF-1α.

Oxidative stress plays an intrinsic role in apoptosis of RGCs. Previous studies showed that bursts of ROS were generated in the retina following ONT, and oxidative stress caused by an imbalance between ROS production and their elimination subsequently induced an irreversible loss of RGCs [2], [4]–[6]. Of note, this study revealed that STC-1 significantly decreased ROS levels in the retina with ONT and in RGCs with CoCl_2_ injury. For the mechanism of STC-1, several studies suggested that STC-1 up-regulated the expression of mitochondrial UCP-2 to uncouple oxidative phosphorylation and thereby diminished superoxide generation [11], [29]. However, UCP-2 was not increased in the retina or in RGCs after STC-1 treatment in this study. Therefore, the mechanism by which STC-1 lowers ROS in RGCs remains to be clarified although the primary effect of STC-1 was probably to decrease apoptosis by reducing oxidative stress.

One time injection of STC-1 was not effective in decreasing apoptosis at 28 days after injury. Considering RGCs undergo apoptosis over 2 weeks after complete transection of an optic nerve, one time injection of recombinant STC-1 may not be sufficient to completely block RGC apoptosis. Multiple intravitreal injections of STC-1 may be necessary for long-lasting effects and are feasible in human patients.

Together, the results demonstrated that STC-1 decreased apoptosis and oxidative stress in RGCs and in the retina. These findings suggest that intravitreal injection of STC-1 may be a promising candidate for treatment of optic neuropathy including glaucoma which is the second most common cause of blindness [3]. Glaucoma is a chronic neurodegenerative disease and characterized by gradual and irreversible loss of RGCs mainly through apoptosis [2]. Strategies to treat this condition are either to prevent RGCs from apoptosis or to stimulate regeneration of axons. Moreover, multiple intravitreal injections of STC-1 are feasible in patients. Therefore, intravitreal injection of STC-1 is particularly attractive for treating chronic diseases such as glaucoma.

## Supporting Information

Figure S1 Gene expression profiles in the retina at days 1 and 7 after optic nerve transection. * p<0.05.
(TIF)
