# Supplementary figures and images for: Stanniocalcin-1 Protects Retinal Ganglion Cells by Inhibiting Apoptosis and Oxidative Damage
Source: PLoS One. 2013 May 7;8(5):e63749. doi: 10.1371/journal.pone.0063749 (PMC3646795; doi:10.1371/journal.pone.0063749)

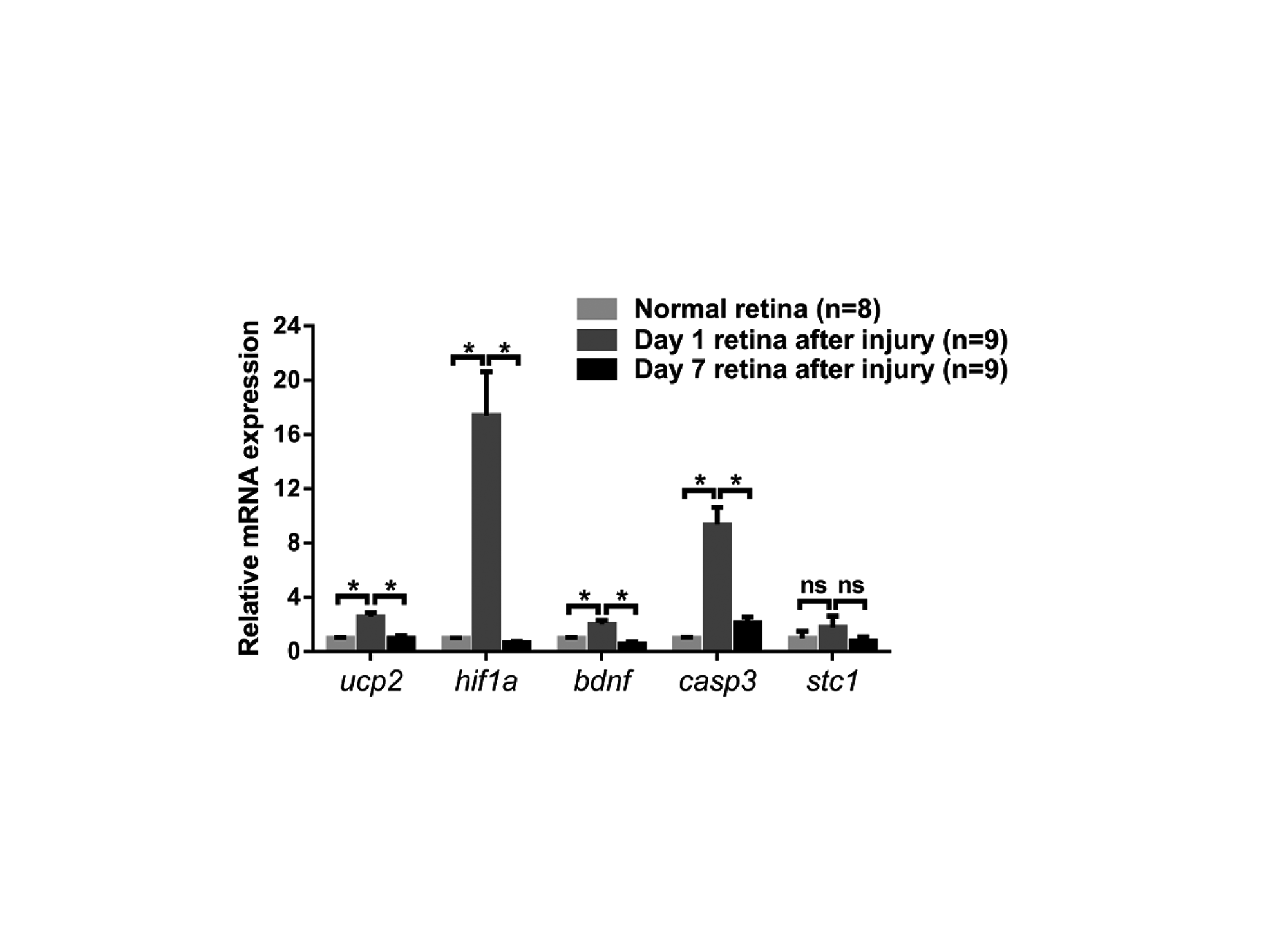

Supplement: Figure S1 — Gene expression profiles in the retina at days 1 and 7 after optic nerve transection. * p<0.05. (TIF) [file pone.0063749.s001.tif]
